# Editorial: Impact of novel omic technologies on biological control against plant pathogens

**DOI:** 10.3389/fmicb.2023.1162422

**Published:** 2023-04-06

**Authors:** Inmaculada Larena, Eduardo A. Espeso, Javier Veloso

**Affiliations:** ^1^Departamento de Protección Vegetal, Instituto Nacional de Investigación y Tecnología Agraria y Alimentaria-Consejo Superior de Investigaciones Científicas (INIA-CSIC), Madrid, Spain; ^2^Departamento de Biología Celular y Molecular, Centro de Investigaciones Biológicas Margarita Salas-Consejo Superior de Investigaciones Científica (CSIC), Madrid, Spain; ^3^Departamento de Biología Funcional, Universidad de Santiago de Compostela, Escuela Politécnica Superior de Ingeniería, Lugo, Spain

**Keywords:** biological control agent (BCA), bio-pesticides, biocontrol, omic-technologies, plant pathogens

Control of plant diseases is mainly achieved by applying chemical pesticides to the crops. Although these chemical treatments have greatly contributed to spectacular improvements in food production and crop yields, their indiscriminate use causes significant environmental damage such as water pollution, soil contamination, increased pathogen resistance and loss of biodiversity, among others. Nowadays, strict regulations control the extensive use of chemical pesticides, and there is increasing the public pressure to remove the most hazardous chemicals from the market. Consequently, the use of biological control (BC) through the application of microorganisms as biological control agents (BCA) or bio-pesticides to reduce plant diseases has emerged as a low-cost, environmentally friendly and sustainable alternative to chemical disease control. However, biocontrol approaches are not as widely adapted as chemical pesticides. Thus, it becomes essential to progress, among many other aspects, in a better understanding of the mechanisms governing biocontrol mediated by microorganisms to improve the efficacy and robustness of treatments. A new generation of molecular technologies has recently provided a powerful approach to better understand the relationship of BCA-host plant-pathogen-environment. These new technologies are known under the term “omics” and include techniques such as micro and macroarrays, next generation sequencing (NGS) technology, proteomics, metabolomics, genomics (including its derivatives pangenomics and metagenomics), and transcriptomics, among others. This Research Topic collects different strategies to better understand BCA-host-pathogen-environment interactions. The use of different omics approaches and synthetic biology, and integrating them with traditional technologies, may thus accelerate the development of BCAs against plant pathogens.

This topic includes manuscripts that focus on new approaches leading to a more successful selection of potential BCAs. Microbiome analysis allow for the discovery of new BCAs. In this sense, Ciancio et al. describe a metabarcoding study to examine the soil/root microbiota (bacteria, fungi, and nematodes) of banana, across several farms with different locations and cultivation techniques. The relationship between the microbiota and these factors is highly relevant in the development of pest control strategies. Anguita-Maeso et al. characterized the xylem sap microbial communities in almond trees, identifying microorganisms that would be good candidates to produce almond plants more resilient to *Xylella fastidiosa* infection. Also, Zhang et al. studied the microbial communities in two cultivars of tea plants, a resistant and a susceptible cultivar. They observed that the relative abundance of *Penicillium* was significantly different between the susceptible and resistant plants, and identified *Penicillium* as a potential biomarker. Zhang et al. analyzed the secondary metabolites produced by resistant and susceptible plants and correlated this with the microbiome. In brief, the authors observed that Penicillium correlated with the secondary metabolite quercetin among others.

Increasing our knowledge of the biocontrol mechanism of BCAs is critical for the subsequent development of appropriate formulations and optimal application timing and methods. Thus, omics disciplines are among the key tools that have significantly improved our understanding of the action mode of BCAs against plant pathogens. Here Ye et al. identified a potential BCA against *Meloidogyne graminicola* which employs multiple anti-nematode mechanisms, including triggering the expression of resistance-related genes and defense enzyme activity to enhance plant resistance. Other works identified the genetic basis of biofilm production based on poly-γ-glutamic acid (γ-PGA) by *Bacillus atrophaeus* NX-12. By generation of a strain lacking the biosynthetic γ-PGA cluster they correlated the formation of biofilm with the colonization of rhizosphere and the biocontrol activity exhibited by this bacteria (Xue et al.).

In the interaction of BCA with the host and the pathogen it is also very important to clarify the mode of action of the target pathogen. Li et al. identified potential genes in the pathogen *Rhizoctonia solani* involved in the production of metabolites and extracellular proteins. This basidiomycete produces a large number of potentially secreted enzymes and small proteins with a putative function as effectors involved in virulence. The reverse genetics and transcriptomic analysis allowed to Lu et al. to explain the role of mating in the virulence of the fungus *Sporisorium scitamineum*. This basidiomycete is the causative agent of sugarcane smut disease in which the formation of dikaryotic strains is essential for filamentous growth and infection in sugarcane plants.

High-throughput analyses are fundamental to the study of the complex tripartite interaction of BCA, host and pathogen. Requena et al. used NGS to compare at the genomic level two strains of *Penicillium rubens*, S27 and PO212. PO212 is an effective BCA against a large number of fungal plant pathogens that infect different horticultural crops while S27 lacks this biocontrol capacity. Comparative genomics showed that PO212 and S27 have a high genomic similarity in gene content. Requena et al. points out the importance to complement this genomic approach with a transcriptomic approach to explain the high similarity in gene sequence but different phenotype. Similarly, Moshe et al. used comparative genomics to study the biocontrol potential of several *Bacillus* strains. The *Bacillus* strains showed different *in-vitro* antagonism against three plant pathogens, *Pythium, Rhizoctonia* and *Fusarium*. The antagonistic effect depended on unique secondary metabolite and chitinase-encoding genes in each Bacillus strain discovered in the comparative genome approach. In this line, Ma et al. use comparative genomic analysis to study two *formae speciales* of *Setosphaeria turcica*. In this regard, some pathogens might act as BCAs in an incompatible host. *S. turcica* f. sp. *zeae* and *S. turcica* f. sp. *sorgh*i cause northern leaf blight disease of corn and sorghum, respectively. In this study, *S. turcica* f. sp. *zeae* was predicted to have fewer secreted proteins, pathogen-host interaction (PHI) genes and carbohydrate-active enzymes (CAZys) than *S. turcica* f. sp. *sorghi* but there were eight effector protein-encoding genes specifically in *S. turcica* f. sp. *zeae*, among which cellulase genes had a major role in pathogenicity.

These contributions highlight the progress in the field of BCA research and its potential to bring solutions from the laboratory to the farm. They also highlight the still unanswered questions about BCA-plant-pathogen-environment interactions and thus provide opportunities for continued research. We hope that the information provided in this topic will be helpful to scientists and students.

## Author contributions

All authors listed have made a substantial, direct, and intellectual contribution to the work and approved it for publication.

